# Supplementary material for: Surface properties of atomically flat poly-crystalline SrTiO3
Source: Sci Rep. 2015 Mar 6;5:8822. doi: 10.1038/srep08822 (PMC4351548; doi:10.1038/srep08822)
Supplement: Supplementary Information [file srep08822-s1.pdf]

Supplementary Information

Surface properties of atomically flat poly-crystalline $SrTiO_3$

Sungmin Woo[1], Hoidong Jeong[1], Sang A Lee[1,2], Hosung Seo[3], Morgane Lacotte[4], Adrian David[4], Hyun You Kim[5], Wilfrid Prellier[4], Yunseok Kim[3], Woo Seok Choi[1*]

[1]Department of Physics, Sungkyunkwan University, Suwon, 440-746, Korea

[2]Insitute of Basic Science, Sungkyunkwan University, Suwon, 440-746, Korea

[3]School of Advanced Materials Science and Engineering, Sungkyunkwan University, Suwon, 440-746, Korea

[4]Laboratorie CRISMAT, CNRS UMR 6508, ENSICAEN, Normandie Universite, 6 Bd Marechal Juin, F-14050 Caen Cedex 4, France

[5]Department of Nanomaterials Engineering, Chungnam National University, Daejeon, 305-764, Korea

[*]e-mail: choiws@skku.edu.

Computational Details

Density functional theory calculations

For surface energy calculation for (100), (110), and (111) facets of STO, we performed spin-polarized density functional theory (DFT) calculations with the VASP code14 and the PBE15 exchange-correlation functional. In order to treat the highly localized Ti 3*d*-orbital, DFT+*U* formalism[1] with U_{eff} = 4.5 eV[2] was applied. The interaction between the ionic core and the valence electrons was described by the projector augmented wave method,[3] and the valence electrons with a plane wave basis up to an energy cutoff of 400 eV. The Brillouin zone was sampled at the Γ-point. The convergence criteria for the electronic structure and the geometry were 10^{-4} eV and 0.03 eV/Å, respectively. We used the Gaussian smearing method with a finite temperature width of 0.05 eV in order to improve convergence of states near the Fermi level.

STO surface modelling and surface energy calculation

The (100) and (110) surfaces were modeled with flat slabs with 9 atomic layers and the (111) surface was modeld with a slab with 11 atomic layers. All layers were fully optimized upon the calculation of the relaxation energy. In each case, two different surface terminations were considered: the TiO_2- and SrO-terminated slabs for the (100) surface, the $SrTiO_3$- and O-terminated slabs for the (110) surface, and the Ti- and SrO_3-terminated slabs for the (111) surface (Table 1). Because both terminations simultaneously appear in the slab model upon cleavage of a $SrTiO_3$ bulk crystal, we calculate the cleavage energy of the $SrTiO_3$ facet, E_{cleavage}, and equally distributed to both terminations. For example, the unrelaxed internal cleavage energy of the (100) surface was calculated as follows:[4, 5]

$$E_{\text{cleavage}}^{(100)} = \frac{1}{4}\left[E_{\text{slab}}^{(100)\text{-TiO}_2} + E_{\text{slab}}^{(100)\text{-SrO}} - xE_{\text{bulk}}\right]$$

where $E_{\text{slab}}^{(100)\text{-TiO}_2}$ and $E_{\text{slab}}^{(100)\text{-SrO}}$ are the energy of unrelaxed TiO_2- and SrO-terminated surfaces, E_{bulk} is the energy of a $SrTiO_3$ unit cell, and x represents the amount of total unit cells in both slabs. For the (100), (110), and (111) surfaces, x was equal to 81, 81, and 99, respectively.

Later, the surface energy of the SrO-terminated (100) facet was calculated as follows:

$$E_{\text{surface}}^{(100)\text{-SrO}} = E_{\text{cleavage}}^{(100)} + \frac{1}{2}\Delta E_{\text{Relaxation}}^{(100)\text{-SrO}},$$

where $\Delta E_{\text{Relax}}^{(100)\text{-SrO}}$ represents the energy released upon the relaxation of the slab. The surface energy of the other studied surfaces was calculated with corresponding E_{slab}, $\Delta E_{\text{relaxation}}$, and x values.

EBSD Analyses of Poly-Crystalline $SrTiO_3$

We obtained detailed orientation information by using EBSD, for the grains selected in Figure 4. **Figure S1** and **Table S1** shows the gradiational inverse pole figure and (h, k, l) values (Miller-indexes), respectively. We note that the (h, k, l) values are mostly large fractional numbers, indicating that the surface orientations are truely random. Moreover, the (h, k, l) values are rather unrelated to the surface properties, and are impossible to intuitivley correlate with the representative orientations. For example, domain (a) and (e) are indeed close to the (100) surface according to the inverse pole figure shown in Figure S1. However, this information is much more difficult to obtain by only looking at the (h, k, l) values shown in Table 1.

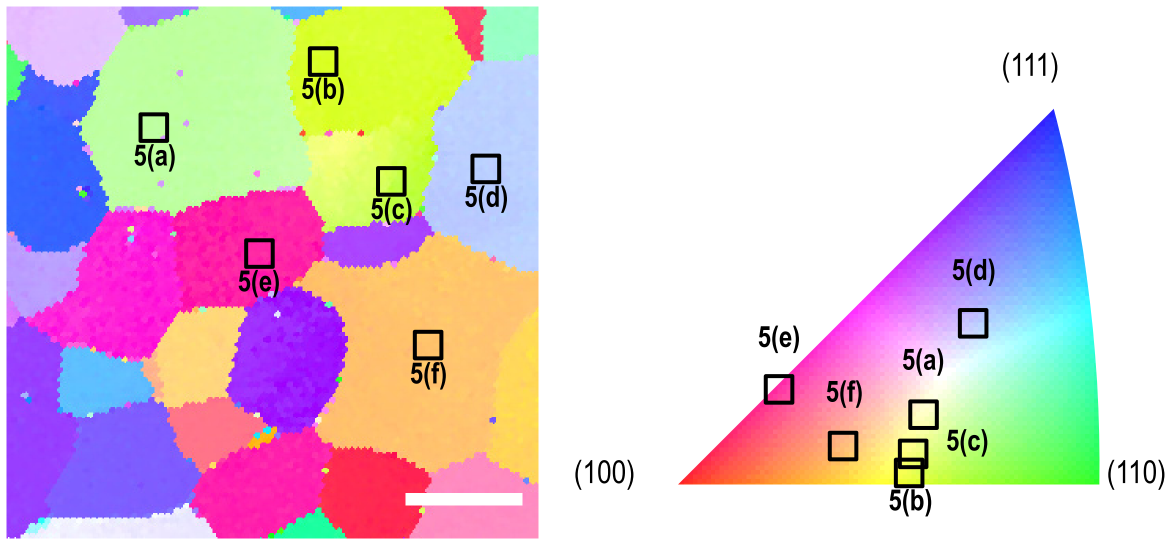

Figure S1. The corresponding domains of each orientation with invers pole figure image. The scale bar indicates 10 μm.

Domain	(h, k, l)
(a)	(246.3, 147.1, 166.6)
(b)	(251.5, 153.5, 181.4)
(c)	(88.0, 92.3, 27.0)
(d)	(246.6, 105.6, 236.5)
(e)	(133.5, 16.5, 132.8)
(f)	(330.7, 109.4, 356.2)

Table S1. The Miller-index of the poly-crystalline domains. Each index is produced by OIM Analysis 5.31

1 Dudarev, S. L., Botton, G. A., Savrasov, S. Y., Humphreys, C. J. & Sutton, A. P. Electron-energy-loss spectra and the structural stability of nickel oxide:An LSDA+U study. *Phys. Rev. B* **57**, 1505-1509 (1998).

2 Park, J. B. *et al.* High catalytic activity of Au/CeO_x/TiO_2(110) controlled by the nature of the mixed-metal oxide at the nanometer level. *PNAS* **106**, 4975-4980 (2009).

3 Blöchl, P. E. Projector augmented-wave method. *Phys. Rev. B* **50**, 17953-17979 (1994).

4 Heifets, E., Goddard, W., Kotomin, E., Eglitis, R. & Borstel, G. Ab initio calculations of the $SrTiO_3$ (110) polar surface. *Phys. Rev. B* **69,** 0163-1829 (2004).

5 Eglitis, R. I. & Vanderbilt, D. First-principles calculations of atomic and electronic structure of (001) and (011) surfaces. *Phys. Rev. B* **77**, 195408 (2008).
